# Label-free analysis of tenofovir delivery to vaginal tissue using co-registered confocal Raman spectroscopy and optical coherence tomography

**DOI:** 10.1371/journal.pone.0185633

**Published:** 2017-09-29

**Authors:** Oranat Chuchuen, Jason R. Maher, Marcus H. Henderson, Michael Desoto, Lisa C. Rohan, Adam Wax, David F. Katz

**Affiliations:** 1 Department of Biotechnology, Faculty of Technology, Khon Kaen University, Khon Kaen, Thailand; 2 Department of Biomedical Engineering, Duke University, Durham, North Carolina, United States of America; 3 Department of Pharmaceutical Sciences, School of Pharmacy, University of Pittsburgh, Pittsburgh, Pennsylvania, United States of America; 4 Magee-Womens Research Institute, Pittsburgh, Pennsylvania, United States of America; 5 Department of Obstetrics and Gynecology, Duke University, Durham, North Carolina, United States of America; Harvard Medical School, UNITED STATES

## Abstract

Vaginally applied microbicide products offer a female-controlled strategy for preventing sexual transmission of HIV. Microbicide transport processes are central to their functioning, and there is a clear need for a better understanding of them. To contribute to that end, we developed an assay to analyze mass transport rates of microbicide molecules within the epithelial and stromal layers of polarized vaginal mucosal tissue during contact with a gel vehicle. The assay utilizes a new diffusion chamber mounted in a custom instrument that combines confocal Raman spectroscopy and optical coherence tomography. This measures depth-resolved microbicide concentration distributions within epithelium and stroma. Data for a tenofovir gel were fitted with a compartmental diffusion model to obtain fundamental transport properties: the molecular diffusion and partition coefficients in different compartments. Diffusion coefficients in epithelium and stroma were computed to be 6.10 ± 2.12 x 10^−8^ and 4.52 ± 1.86 x 10^−7^ cm^2^/sec, respectively. The partition coefficients between epithelium and gel and between stroma and epithelium were found to be 0.53 ± 0.15 and 1.17 ± 0.16, respectively. These drug transport parameters are salient in governing the drug delivery performance of different drug and gel vehicle systems. They can be used to contrast drugs and vehicles during product design, development and screening. They are critical inputs to deterministic transport models that predict the gels’ pharmacokinetic performance, which can guide improved design of products and optimization of their dosing regimens.

## Introduction

Microbicides are virus-neutralizing molecules used in biomedical products being developed for women and men to protect themselves against sexual transmission of Human Immunodeficiency Virus (HIV) and other pathogens [1]. Development of antiretroviral microbicide products, such as the tenofovir gel [2, 3] and dapivirine intravaginal ring (IVR) [4], has been a key focus in the microbicide research field. A microbicide gel can coat the vaginal or rectal walls, creating a thin protective layer that retards HIV virion migration from semen to mucosal surfaces [5], and delivering an anti-HIV drug into the mucosal tissue layers to provide prophylactic protection against HIV infection of host cells therein.

At present no microbicide gel has successfully demonstrated efficacy in sequential Phase III clinical trials. Although 1% tenofovir gel effectively reduced HIV sexual transmission in women by 39% in a first phase III study (CAPRISA 004) [2], it failed to demonstrate efficacy in its second (VOICE, MTN003) [6] and third efficacy trials (FACTS 001) [7]. The failures in those latter two trials were associated with poor subject adherence [8, 9]. This may have been due, in part, to the messiness of the relatively large applied gel volume (4 mL) and the demands of its dosage regimen. If gel volume could be reduced, and a more user-friendly dosage regime practiced, adherence to use could be improved. Improved understanding of mechanisms of how gel volume and dosage regimen govern microbicide pharmacokinetics and consequent pharmacodynamics will clearly contribute to this. The work here contributes to that understanding.

*In vivo*, microbicide gel molecules migrate out from the gel and into/through adjoining vaginal fluids and mucosal tissue. The human vaginal mucosa has two layers: an upper stratified squamous epithelium (about 100–200 μm thick); and a lower lamina propria (stroma), which is about 3 mm thick and is composed largely of connective tissue. HIV-infectible host cells reside primarily within the lamina propria, and both HIV virions and anti-retroviral drugs must thus migrate some distance down from the lumen before attaining them. The mass transport rates of molecules within and between the vaginal gel, epithelial and stromal layers are biophysically coupled [10, 11].

*In vitro* assays seek to capture and characterize gel release and mucosal transport of microbicide molecules in pharmacologically meaningful ways. A common configuration is embodied in a Franz Cell apparatus [12]. It measures the cumulative amount of drug permeating through tissue in a fluid receptor compartment vs. time [13, 14]. The drug concentration-versus-time curve is typically linearized to compute an effective permeability coefficient, P. For steady state drug diffusion through a homogeneous tissue layer (thickness h), P = kD/h, where k is the drug partition coefficient at the vehicle-tissue interface, and D is the drug diffusion coefficient within the tissue layer. However, if the tissue contains layers with different structures, then the diffusion coefficients of a molecule within each of those layers may be different. Thus, the permeability is a lumped parameter that does not reveal this distinction. Moreover, if there are solubility differences between the drug in its vehicle and the underlying tissue, then the partition coefficient k may not be unity, at the gel-epithelium interface and/or at the interface between the epithelium and stroma. Consequences of these distinctions have been elaborated in deterministic compartmental modeling of tenofovir transport from a gel layer into vaginal mucosal tissue [11, 15, 16]. These modeling analyses have indicated that differences in diffusion coefficients between the two mucosal layers, together with the different thicknesses of those layers, lead to non-uniform drug distributions within and between the layers.

The non-uniform distributions in epithelium vs. stroma, and partitioning at interfaces, limit interpretations of biopsies collected in *in vivo* pharmacokinetic (PK) studies. In clinical PK studies, biopsy specimens obtained after drug application are typically homogenized and the weight-averaged tissue concentrations are obtained by traditional methods, e.g., liquid chromatography coupled with tandem mass spectrometry (LC-MS/MS) [2, 17, 18]. These standardized methods are highly sensitive, but they are sophisticated to conduct and provide only mass- averaged tissue concentration vs. time data in the combined epithelial plus stromal tissues. The deterministic PK models show that concentrations in vaginal biopsies overestimate tenofovir concentrations in the vaginal stroma (location of HIV infectible cells) by as much as one order of magnitude in an uncontrolled manner [10, 16]. Thus, measurements in biopsies may not yield incisive understanding of how rapidly and completely the test drugs are transported into the target mucosal tissue, and of drug concentrations at the stromal sites where they act.

Non-invasive methods are attractive as they offer rapid measurements with minimal sample perturbation, and can help fill this critical gap in the microbicide field. Fluorescence microcopy and Fourier transform infrared (FTIR) spectroscopy have been used for non-invasive diagnosis of biological samples and diseased tissues [19, 20]. However, intrinsic tissue fluorescence limits the use of fluorescence microscopy to detect target analytes within tissue. Since water is a very strong IR absorber, FTIR is not appropriate for analysis of analytes in hydrated or *ex vivo* tissue samples that contain water.

Confocal Raman spectroscopy (RS) is a chemically-specific optical method that can quantify depth-dependent concentration distributions of target analytes in *ex vivo* tissue specimens [21–24]. Tissue autofluorescence and photobleaching can be mitigated by utilizing a suitable excitation source such as a near-infrared diode laser. The shifts in energy of Raman scattered light relative to the incident light correspond to energy differences between the vibrational states of a sample. Thus, these Raman shifts are characteristic of specific chemical bonds and can be analyzed to determine the chemical composition of the material. Optical coherence tomography (OCT) is a noninvasive imaging technique that detects depth-resolved reflections of light to yield cross-sectional images of subsurface tissue microstructures on the micron scale [25, 26]. As opposed to traditional invasive methods that employ histopathology, OCT non-destructively provides an ‘optical biopsy’ that reveals details about tissue morphology in real time without tissue removal [27].

Coherent RS has also been used to non-invasively evaluate drug delivery [28]. Although coherent Raman techniques offer significant improvements in acquisition speed, the range of wavenumber shifts and therefore the number of molecular vibrations that can be measured is typically less than that measured by spontaneous, confocal RS [29]. Because high acquisition speeds were not required for our study, we chose to utilize spontaneous, confocal RS. In our combined confocal RS and OCT instrument [24], confocal RS and OCT measurements are co-registered. This enables drug concentrations to be quantified in different tissue layers by using OCT to distinguish each layer and confocal RS to determine the drug concentration within the layer. Here, the methodology was applied to measure the transport of tenofovir, a leading microbicide drug, from a microbicide gel into vaginal mucosal tissue. A tenofovir microbicide gel was applied to tissue epithelial surfaces and measurements were taken as the drug diffused into the epithelium and stroma of the polarized excised vaginal tissue specimens contained within a new drug diffusion chamber. Concentration distributions were fitted with a time-dependent, multi-compartmental diffusion model to derive the drug partition and diffusion coefficients in epithelium and stroma. Collectively, this new assay provides a tool for improving our understanding of anti-HIV drug delivery to mucosal tissue.

## Materials and methods

### Ethics statement

This study used excised porcine vaginal tissues. Porcine female reproductive tracts were harvested immediately after slaughter with permission from a local abattoir (Hatley Family Farm, Hurdle Mills, NC) as in our previous studies [23].

### Tissue harvesting, storage and specimen preparation

Porcine vaginal tissue is used as a surrogate for human vaginal tissue. It has been shown to demonstrate excellent correlation with human vaginal tissue in histology and structure, composition, as well as permeability characteristics towards water and hydrophilic molecules [30, 31]. Fresh porcine female reproductive tracts were harvested from domestic pigs and transferred to our laboratory immediately after sacrifice. The tracts were transported in ice-cold isotonic, carbogen-gassed (5% CO_2_ and 95% O_2_) Krebs-Ringer bicarbonate buffer (pH 7.2; Sigma cat. # K4002: 0.49 mM MgCl_2_, 4.56 mM KCl, 119.8 mM NaCl, 0.70 mM Na_2_HPO_4_, 1.5 mM NaH_2_PO_4_), supplemented with 15 mM NaHCO_3_, and 9.99 mM glucose. Vaginal tissue was excised and excessive connective tissue and fat were removed with surgical scissors. The tissue was snap frozen in liquid nitrogen and stored at -80°C. Studies have shown that snap freezing does not alter the histology and permeability characteristics of vaginal tissue towards different molecules, both hydrophobic and hydrophilic, when used after a single freeze-thaw cycle [30, 32–34].

### Test gel and tissue transport chamber

**Fig 1:**
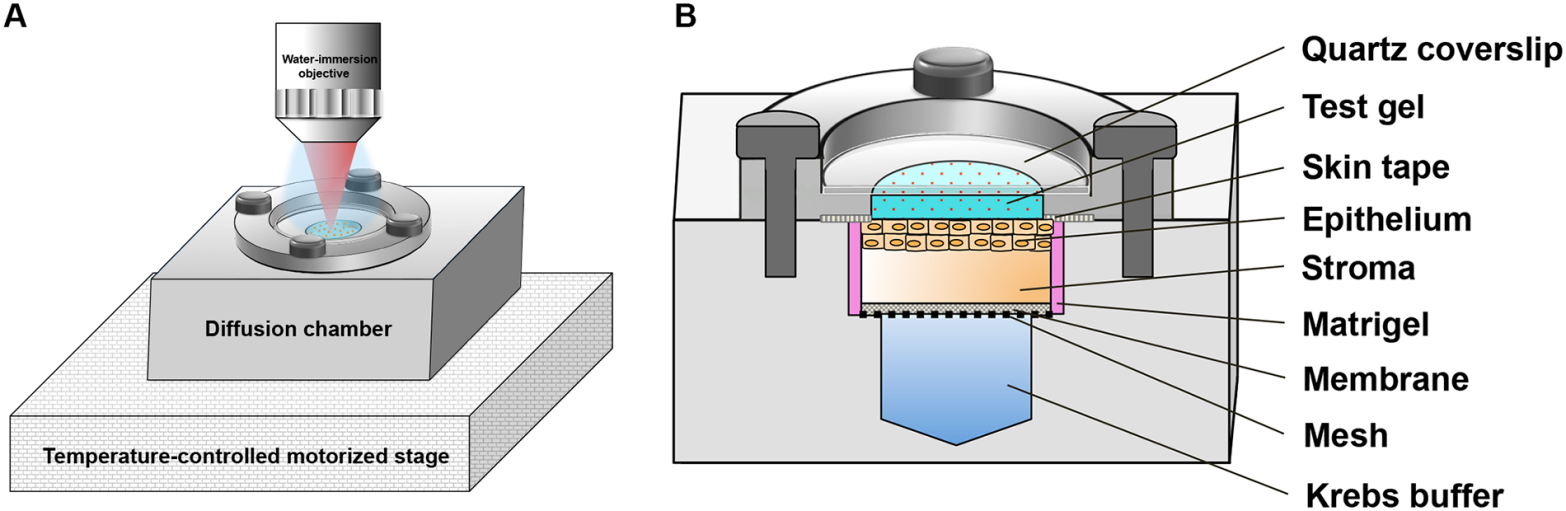
Experimental setup of a transport assay for measurements of drug concentration distributions in tissue. (A) A custom-made tissue chamber was placed on a heated motorized stage in order to maintain the diffusion experiment at 37°C. Measurements were acquired using a water-immersion objective. (B) A schematic cross-sectional view of the sample chamber, showing the components of the chamber.

The test gel was a 2% tenofovir reduced glycerol (RG, 5% glycerol) gel (pH 4–5). Thus, the pH of this gel was designed to simulate the ambient pH in the human vagina. This formulation was a slightly modified version of the clinical 1% tenofovir RG gel [3, 14]. We doubled the tenofovir concentration in the gel in order to increase its concentration in tissue, thereby improving sensitivity of the measurements. A tissue sample chamber (cf. Fig 1) was constructed for standard preparations of *in vitro* diffusion experiments in polarized tissues. An aluminum block was specifically designed and fabricated so that it contained two compartments: a buffer compartment and a tissue compartment. The top piece was an aluminum clamp that was applied over the tissue surface to hold the tissue in place; a hole was cut in the middle so that a gel could be loaded over the tissue surface. For a given experiment, an excised tissue specimen (18 ± 2-mm diameter) was fitted in the tissue compartment (4-mm thickness; 18-mm diameter); the tissue rested on a polyester membrane (3-μm pore size, GE Healthcare Bio-Sciences, Pittsburgh, PA) supported by a mesh. A bath of Krebs buffer supplemented with glucose was loaded underneath in the buffer compartment to nourish the tissue. Before applying the test gel, tissue edges were carefully sealed with Matrigel (BD Biosciences, San Jose, CA) and a waterproof hole-cut skin tape (Blenderm^™^, 3M) to prevent leakage of the gel formulation along the specimen’s sides. The top aluminum piece with a hole cut (1-mm thickness; 14-mm diameter) in the middle was then placed over the tissue surface. Gel was loaded onto the tissue surface; timing of the experiment began at the onset of gel-tissue contact. A quartz coverslip was inserted over the gel surface and fitted into the top aluminum clamp. The coverslip prevented gel hydration and dilution by water droplets that were applied when using a water-immersion objective lens. The chamber was then tightened with four screws to seal it. The chamber preparation was placed on a temperature-controlled motorized stage to maintain the tissue sample at 37 ± 0.5°C during the period of measurement. Six experiments were performed—each used an excised tissue specimen from a different pig. Porcine mucosal tissue has been shown to be viable for up to 6 h following tenofovir gel treatment at 37°C [23]; therefore, each experiment was run up to 6 h.

### Instrumentation

All data in this study were acquired with a locally-constructed confocal Raman spectroscopy and optical coherence tomography instrument integrated with a custom brightfield microscope [24]. The OCT subsystem (axial resolution of 8 μm and a lateral resolution of 28 μm) was used to monitor tissue morphology and to measure the thicknesses of the gel and the epithelial layers of the mucosal tissue. The confocal RS subsystem (axial resolution in air of 20 μm and a lateral resolution of 5 μm) utilizes a 785 nm laser diode (LD785-SH300, Thorlabs Inc., Newton, NJ) for excitation that delivers about 30 mW of power to the sample.

**Fig 2:**
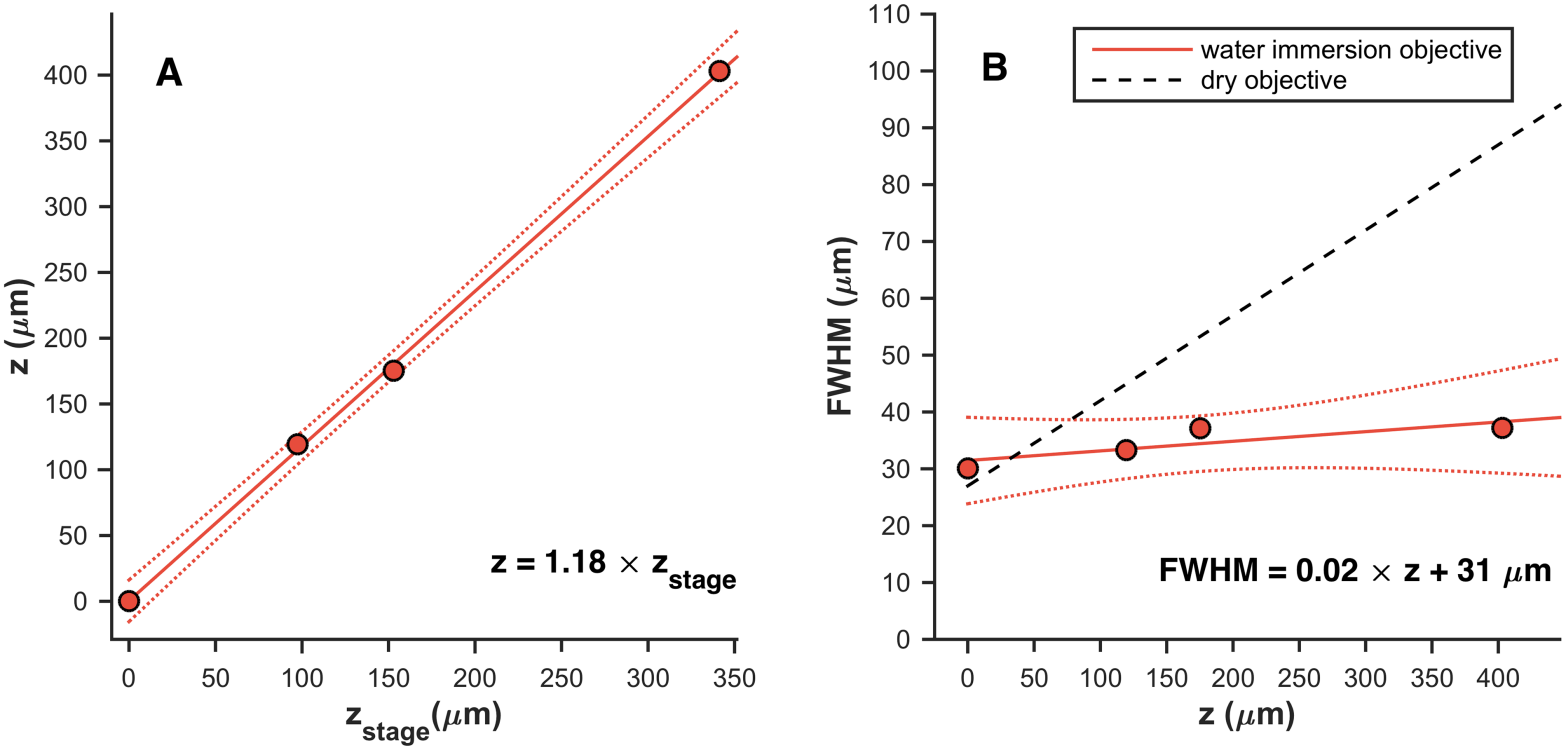
Effects of scattering and spherical aberration on axial resolution and sampling depth. (A) Relationship between the confocal RS true sampling depth, z, and the nominal depth or the stage position, *z*_*stage*_. (B) Relationship between the axial resolution (full-width at half-maximum, FWHM) of the confocal RS subsystem and z. The water immersion objective provided a significant improvement in resolution compared to the dry objective lens [24] for measuring biological tissue. Least-squares fits (solid lines) reveal that both relationships are approximately linear over this range. The dashed lines represent 95% confidence intervals for the fits.

The detailed design and specifications of this multimodal instrument have been described previously [24] with the exception of one modification that was recently introduced to improve performance. The initial design used a long working distance, dry objective lens. This lens induced spherical aberration in the system due to the relatively large refractive index mismatch at the tissue surface. Ultimately, this degraded the resolution and penetration depth. In order to reduce spherical aberration and mitigate the associated deleterious effects [35], the lens was replaced with a water immersion objective lens (W Plan-Apochromat 40x/1.0, Carl Zeiss Microscopy, LLC, Thornwood, NY). Two-layer tissue phantoms were then constructed to characterize the axial resolution of the confocal RS subsystem as a function of penetration depth, as well as the relationship between the stage position *z*_*stage*_ and the true sampling depth *z*, as described previously [24]. The results of these measurements, which highlight the improved performance of the instrument, are displayed in Fig 2. Significantly, the water immersion objective lens provides an axial resolution of approximately 30–40 μm over the entire practical depth range. The relationship between z and *z*_*stage*_ was found to be 1.18. Thus, to calibrate all depths reported in this study, the nominal depth or *z*_*stage*_ was multiplied by this correction factor in order to obtain the true sampling depth within the tissue.

### Preparation of a calibration standard of tenofovir in homogenized tissue

Porcine vaginal tissue was sliced with a Thomas Stadie-Riggs tissue slicer (Thomas Scientific, Swedesboro, NJ) to remove excess stromal connective tissue and achieve approximately 500-μm thick sections of epithelial and upper stromal tissues. The sliced tissues were minced with surgical scissors and loaded into centrifuge tubes preloaded with ceramic beads. The tissue was homogenized using a tissue homogenizer (Bead Ruptor 12 Homogenizer, Omni International Inc., Kennesaw, GA) for 10 cycles of 30 seconds each. After each 30s of homogenization, the tube was chilled on ice to prevent heat build-up in the sample during the bead rupture process. The homogenized tissue was mixed with known amounts of tenofovir. Twelve tissue samples were prepared, each containing a different concentration of tenofovir, ranging from 0.00 to 1.44% w/w. Raman spectra were acquired for each sample and the ratio of the spectral contribution of tenofovir to that of tissue was related to the known tenofovir concentration to generate a calibration curve. The limit of detection (LOD) of tenofovir in tissue was determined from the root mean squared error of prediction (RMSEP), which has been shown to be a reliable estimate of the minimum detectable concentration [36]. The limit of quantification (LOQ) was estimated based upon the variability in the responses of blank tissue specimens. It was computed as mean blank value (n = 11) plus ten times the standard deviation [37].

### Data analysis and derivation of the transport parameters

**Fig 3:**
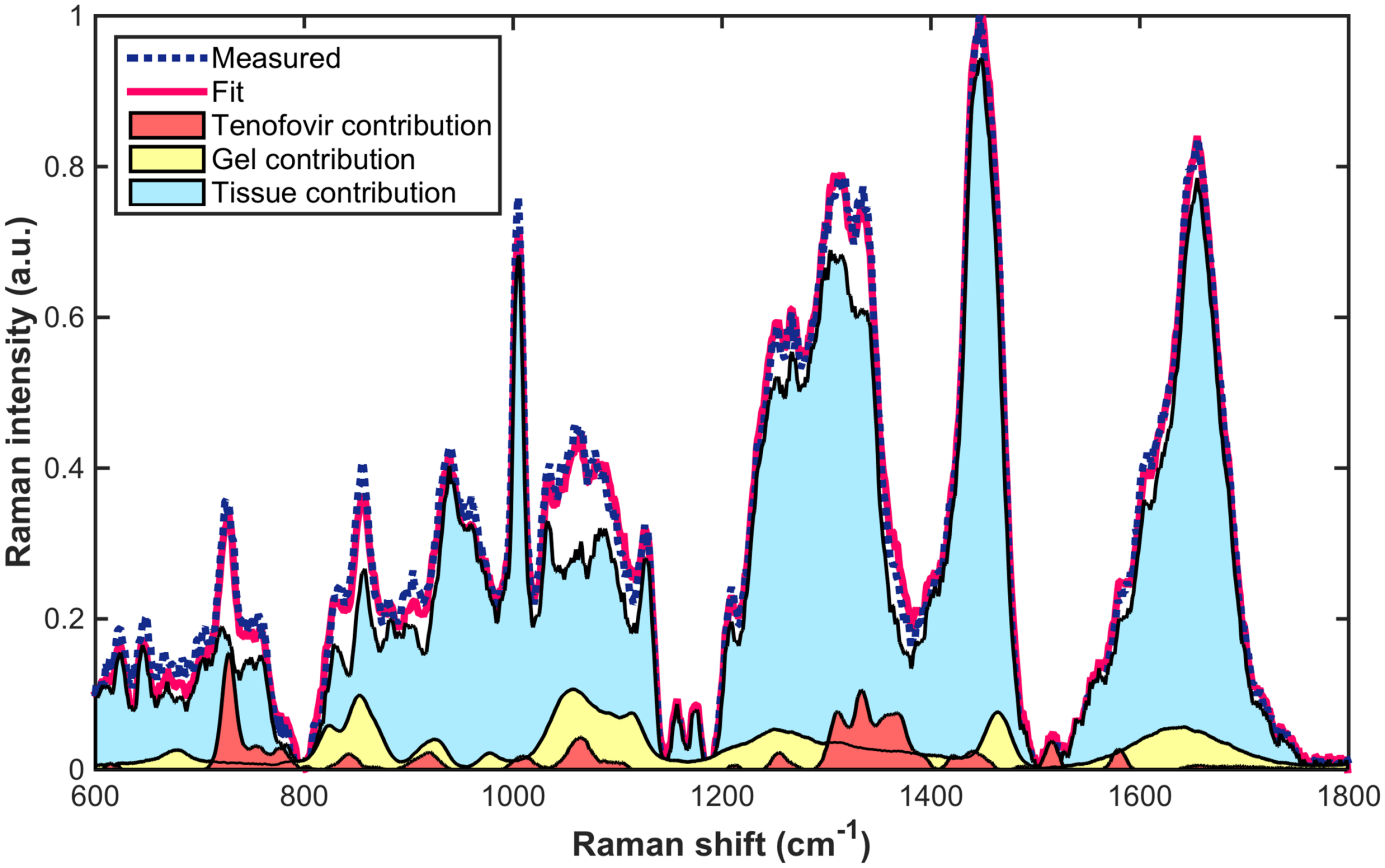
Representative least-squares fit of a Raman spectrum of tenofovir -gel treated tissue from a typical experiment. The tissue was incubated under tenofovir gel in the transport chamber. The spectrum was decomposed by ordinary least squares fitting with basis spectra of tenofovir, gel, and tissue in order to yield the underlying spectral contribution of each component.

Raman spectra were processed with custom software written in MATLAB (R2014b, MathWorks, Natick, MA), as previously described in [24]. The MATLAB codes are freely available by contacting the authors. The measured spectra were analyzed by classical least squares [38] by fitting with a library of pure spectral components including spectra of tenofovir, placebo gel, and porcine vaginal tissue in order to determine the underlying spectral contribution of each component (Fig 3). The ratio of the spectral contribution of tenofovir to that of tissue was converted to concentration values, using the established calibration curve. This quantitative approach of determining drug concentration in tissue was previously validated by our group [24].

**Fig 4:**
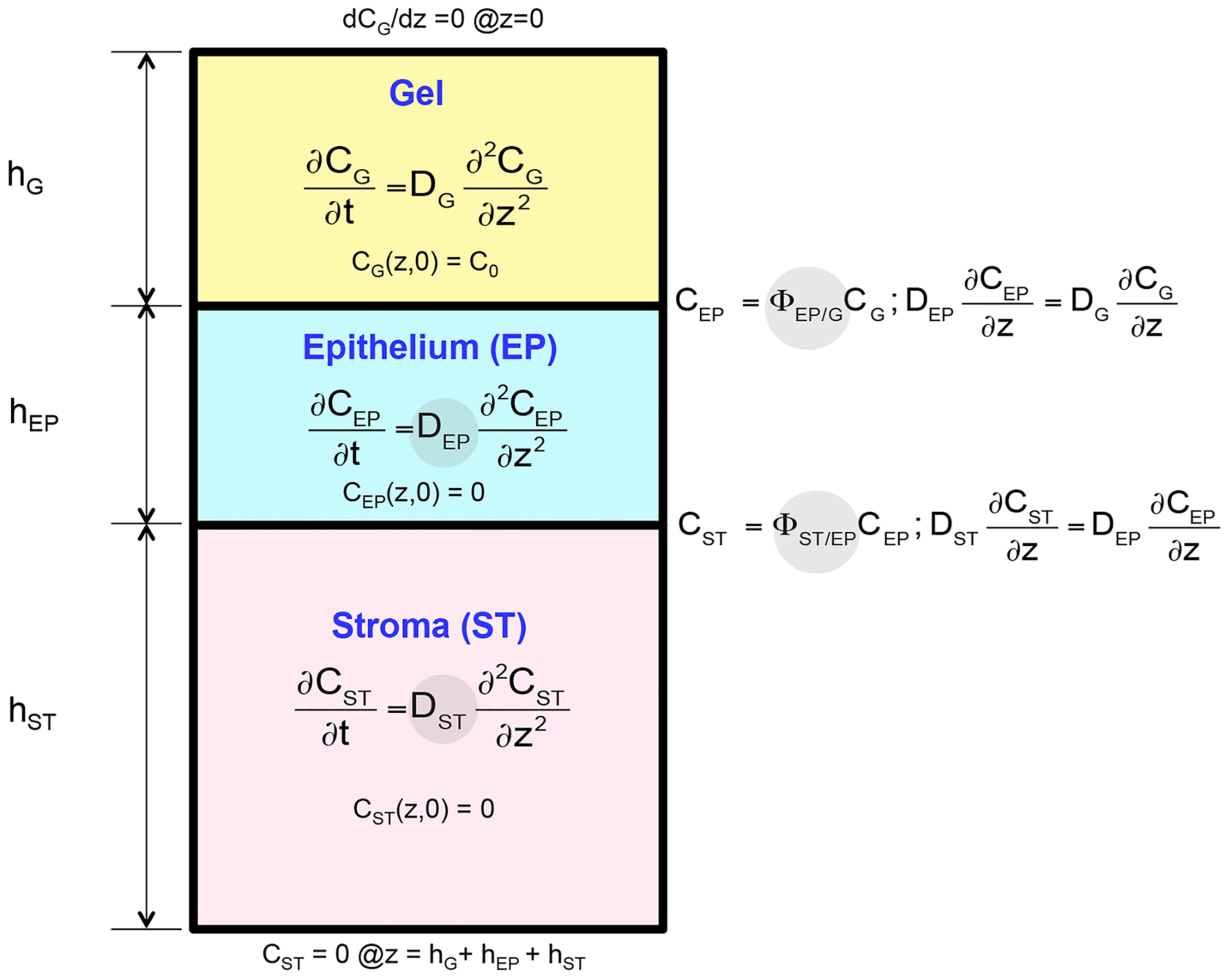
Schematic of drug transport model for tissue transport assay. D_EP_, D_ST_, Φ_EP/G_ and Φ_ST/EP_ were derived from fitting experimental data with the model.

Tenofovir concentration data in tissue over time and space were fitted with a diffusion model with three compartments (Fig 4) using MATLAB. The model was previously described in [10]. Briefly, the partial differential equations (PDEs) were first converted to a system of ordinary differential equations (ODEs) using the forward difference method. The set of ODEs was then solved by a built-in Matlab function, ode15s. Fitting was performed to optimize the transport parameters via nonlinear curve fitting, utilizing a built-in MATLAB toolbox function, lsqnonlin in MATLAB Version R2014b. The function employs a trust-region-reflective least squares algorithm to minimize the sum of squared residuals between the real data and predicted concentration values based on the model.

The four parameters of interest were the diffusion coefficients in epithelium (D_EP_) and stroma (D_ST_), as well as the partition coefficients at the interface between epithelium and gel (Φ_EP/G_) and between stroma and epithelium (Φ_ST/EP_). Other inputs to the model were: the diffusion coefficient in the gel (D_G_), which we measured previously [39], the initial drug concentration (C_o_), as well as the thicknesses of the gel layer (h_G_), epithelium (h_EP_), and stroma (h_ST_). h_G_ and h_EP_ were measured using the OCT subsystem. Since the thickness of the stroma exceeded the penetration depth of the OCT subsystem, h_ST_ was measured using a micrometer.

## Results

### Confocal Raman spectral analysis and measurement sensitivity in tissue

**Fig 5:**
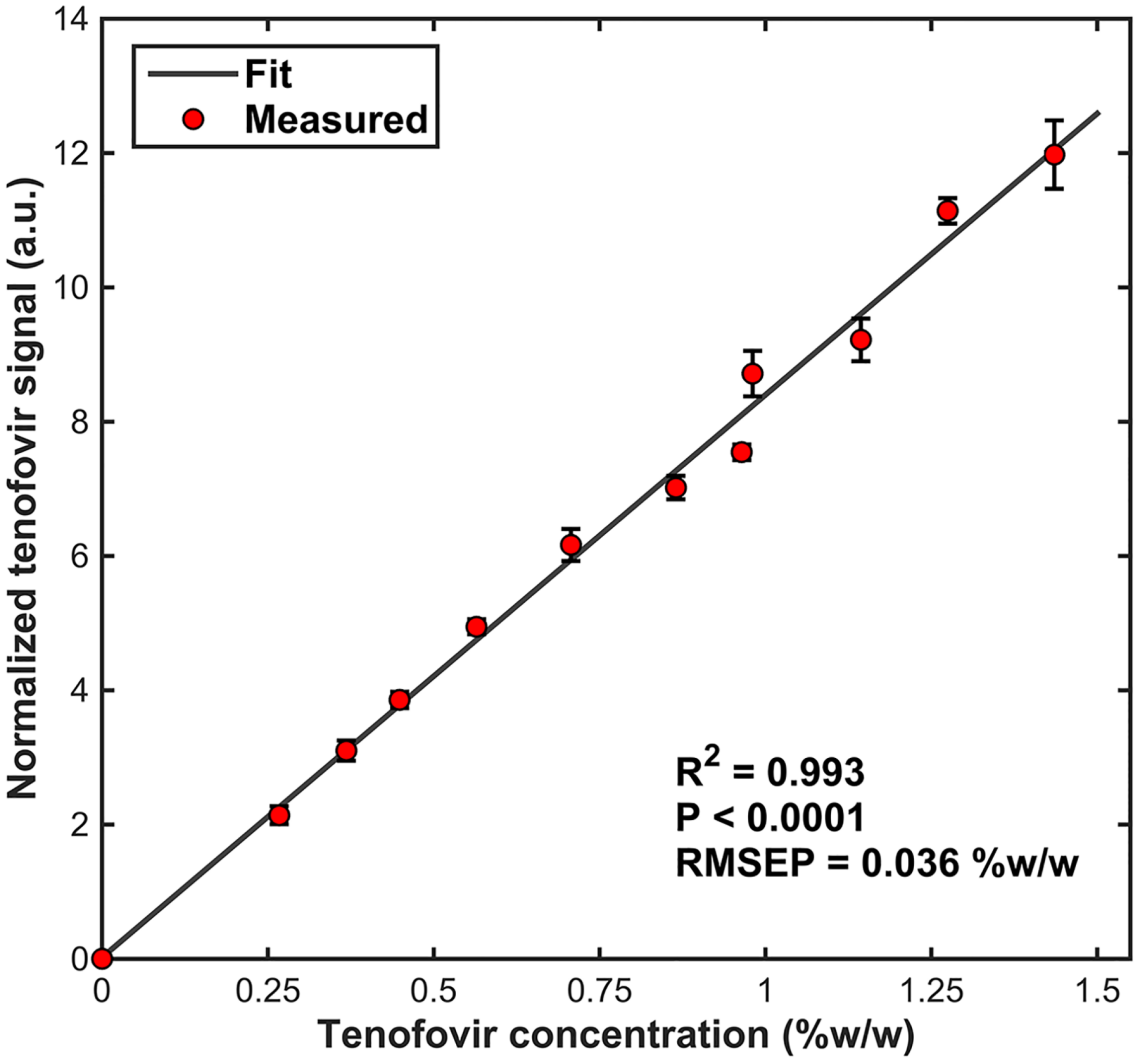
A calibration curve of tenofovir in homogenized tissue for determination of absolute tenofovir concentration. Each data point represents mean ± SE of 5 measurements, except for the control (0%) and 0.45% data points, which represent means of 4 and 6 measurements, respectively (complete data are available in the S1 Table). Error bars are invisible when smaller than the symbols. We observed a strong linear dilution response (R2 ≥ 0.99, P <0.0001) for concentrations of tenofovir in the tissue. The root mean squared error of prediction (RMSEP) represents the limit of detection (i.e., the prediction accuracy of tenofovir concentration in tissue).

A strong linear concentration response (R2 ≥ 0.99) was observed between the Raman measurements of tenofovir in tissue and the corresponding absolute concentrations (Fig 5) as expected for spontaneous Raman scattering. This relationship was used to create a standard curve relating the Raman scattered intensity to the absolute concentration and produced a root mean square error of prediction and limit of detection of 0.036% w/w in the tissue. The limit of quantification was estimated to be 0.095% w/w.

### Co-registered confocal RS and OCT measurements

**Fig 6:**
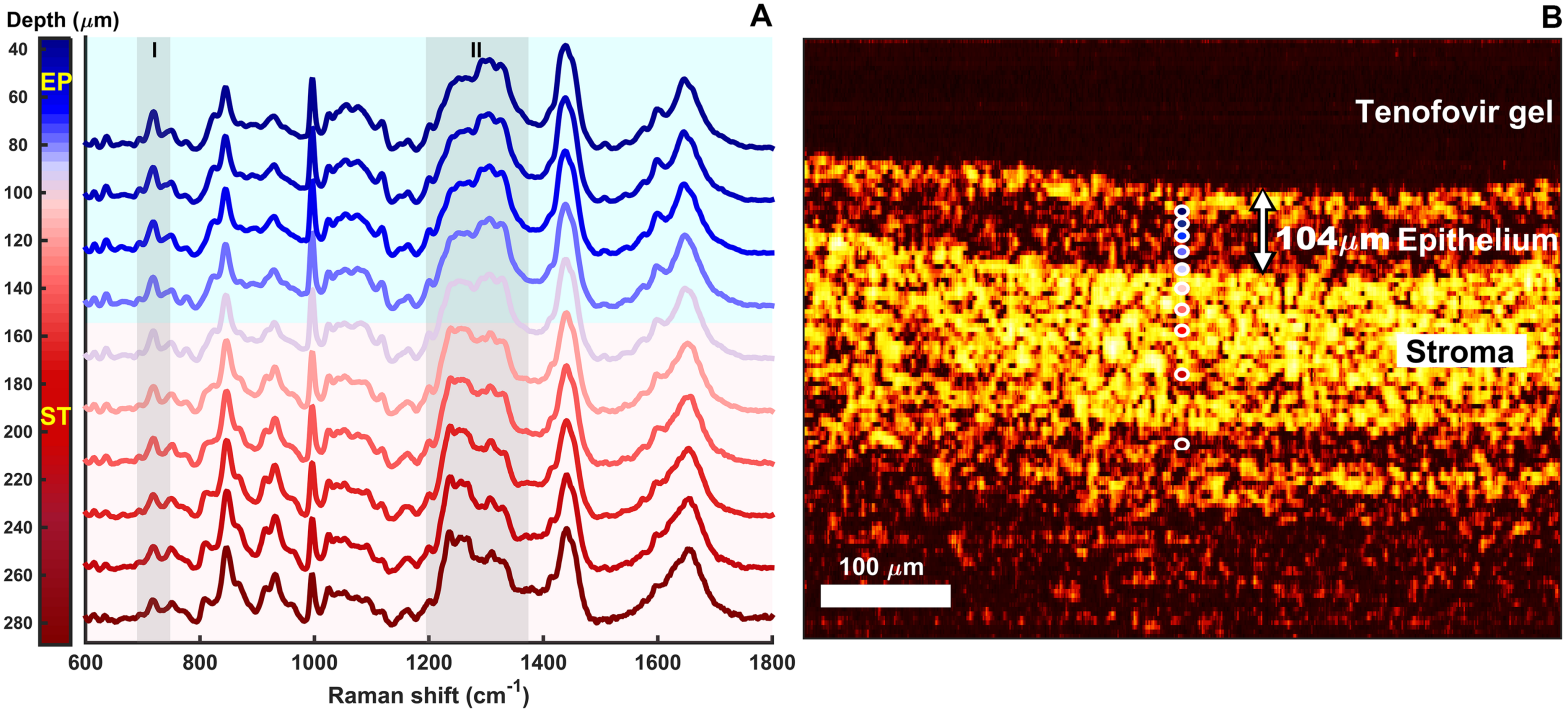
Co-registered confocal RS and OCT measurements of a tenofovir gel—Treated tissue specimen in a diffusion chamber. (A) Spectra are normalized by the mean intensity across all wavenumbers. Tenofovir peak intensities (I) decreased with depth into the tissue, while the tissue band intensities (e.g., at 1440–1450 cm^-1^ –representing CH_2_ band of lipid and protein [40]) remained relatively constant. The spectral changes due to varying amount of collagen content between epithelium and stroma appeared in the amide-3 band region (1200–1400 cm^-1^, II). Spectra are offset for clarity of presentation. (B) A well-defined epithelial layer was observed, supported by the stroma. The gel was transparent and did not exhibit scattering; thus it appeared dark in the OCT image. The Raman spectral changes observed at a depth of approximately 100 microns are consistent with the location of the basement membrane identified by OCT.

An example of co-registered OCT and confocal RS z-scan measurements of a tissue specimen incubated under a layer of the test gel is shown in Fig 6. A well-defined epithelium is seen with OCT, supported by the stroma. The confocal Raman spectra show spectral contributions from tenofovir (e.g., adenine ring mode near 725 cm^-1^ [23], I), which had diffused from the gel down into the tissue. The peak intensities of tenofovir decreased with depth into the tissue, while the tissue peak intensities (e.g., CH_2_ deformation bands near 1440–1450 cm^-1^ [40]) remained relatively unchanged. This suggests a decrease of tenofovir concentrations into the tissue.

Confocal RS reveals spectral differences between epithelium and stroma and the spectroscopic transition corresponds to the epithelial thickness measured with OCT. The spectral difference due to varying amount of collagen content between epithelium and stroma is apparent in the amide-3 mode region at approximately 1200–1400 cm^-1^ (II) [41]. The Raman vibrations at 1246 cm^-1^ and 1271 cm^-1^ correspond to collagen fibrils [42] that are present abundantly in the basement membrane and the stroma.

### Tenofovir transport properties in epithelium versus stroma

**Fig 7:**
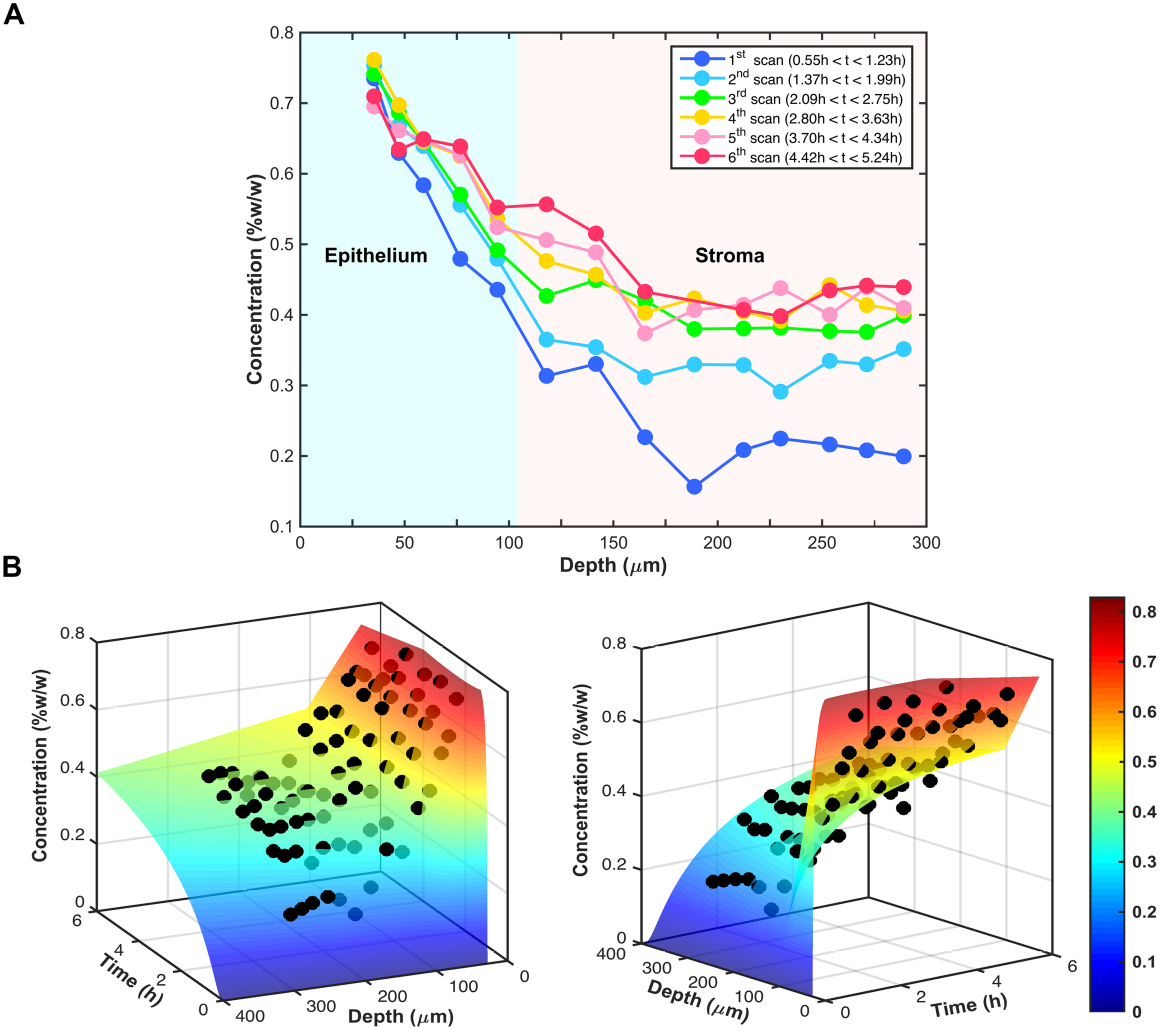
A representative spatiotemporal concentration profile of tenofovir in tissue. The thicknesses of the epithelium (h_EP_), the stroma (h_ST_), and the gel overlayer (h_G_) were 104 μm, 3387 μm, and 1078 μm, respectively. (A) Tenofovir concentration distributions obtained from multiple depth scans. Each data point represents a measurement at a different time and depth. (B) Tenofovir concentration distributions fitted with a drug diffusion model to derive fundamental transport parameters. Best fit parameters were: D_EP_ = 5.04 x 10^−8^ cm^2^/s, D_ST_ = 4.14 x 10^−7^ cm^2^/s, Φ_EP/G_ = 0.57, and Φ_ST/EP_ = 1.01. Data are displayed in two different viewing angles with the best-fit surface that describes tenofovir concentration distributions in the tissue.

Tenofovir concentration distributions varied with time and depth and were different between epithelium and stroma. Fig 7 shows an example of such differences. The concentrations decreased with depth from the epithelium into the stroma. The overall drug diffusion profile tended to be steeper in epithelium than that in stroma (Fig 7A), suggesting a higher transport rate in the stromal compartment. Tenofovir concentration distributions were fitted with a diffusion model of drug transport in order to derive the transport parameters (Fig 7B); these values are summarized in Table 1 (the complete data set is available in the S2 Table).

**Table 1:** Transport parameters derived from six independent tissue diffusion experiments—Each experiment used a different tissue sample (n = 6).

Parameter	Mean ± SD	Range	Median
D_EP_ (cm^2^/s)	6.10 ± 2.12 x 10^−8^	[2.71 x 10^−8^, 8.56 x 10^−8^]	6.11 x 10^−8^
D_ST_ (cm^2^/s)	4.52 ± 1.86 x 10^−7^	[1.22 x 10^−7^, 6.57 x 10^−7^]	4.62 x 10^−7^
Φ_EP/G_	0.53 ± 0.15	[0.38, 0.81]	0.50
Φ_ST/EP_	1.17 ± 0.16	[1.01, 1.41]	1.11
R^2^	0.83 ± 0.07	[0.76, 0.93]	0.83

## Discussion

We have developed and applied an optical assay based on the integrated performance of confocal Raman spectroscopy and optical coherence tomography to analyze microbicide transport from gel vehicles into and through tissue specimens. In our previous studies, we measured drug transport in tissue which was incubated under a gel delivery layer in a Transwell configuration, removing the gel delivery layer, and then using confocal RS to quantify the drug concentration versus depth in each tissue specimen [23, 24]. Each sample was measured at a single time point rather than monitoring changes in the concentration profiles over time. Measurements from multiple specimens (each incubated for a different time) were then combined to generate an average time-dependent concentration profile. We noted the limitations of this approach related to inter-tissue sample variability as well as drug diffusion during the data acquisition period.

Here we present an enhanced methodology that offers two substantial improvements: (1) data are acquired at successive times in each tissue sample while that specimen is maintained under a layer of gel; and (2) the time- and space- dependent concentration data are processed to deduce fundamental drug transport properties for both epithelial and stromal tissue layers. This capability can extend to analysis of other molecules migrating into a range of tissues. The assay provides minimally invasive, comprehensive analysis by optical sectioning within a biologically and pharmacologically relevant matrix. Thus it improves the fidelity of measuring and characterizing drug transport in tissue. This approach to characterizing drug transport is more informative than traditional permeability assays because it quantifies the time and space-dependent drug concentration distribution within the tissue during permeation, and it deduces fundamental layer-specific mass transport parameters, the diffusion and partition coefficients.

Our confocal Raman results showed that tenofovir concentrations in the mucosal tissue were not uniform with depth, not simply because the diffusional concentration profiles had inherent spatial gradients but, also, because tissue structure was not uniform. The results indicate that the epithelium presents a rate limiting barrier for tenofovir transport through vaginal mucosal tissue. Consequences of the tenfold differences in diffusion coefficients for epithelium vs. stroma (Table 1) can be appreciated using the simple formula for the root mean square diffusion distance $L=\sqrt{2Dt}$ where *D* is the diffusion coefficient and *t* is time [43]. This shows that for a given time, a tenfold difference in diffusion coefficient results in a threefold difference in distance diffused. Considered another way, the diffusion time *t* for distance *L* is inversely proportional to the diffusion coefficient and thus increases tenfold as the diffusion coefficient decreases by one order of magnitude. Such predicted and measured (*in vitro*) discrepancies are likely to manifest in biopsies collected in *in vivo* PK studies (simulated in our modeling studies [10, 11]).

Difference in structural properties between epithelium and stroma likely contributes to the difference in diffusion coefficients between vaginal epithelium and stroma. The porcine and human vaginal epithelia are stratified squamous, containing a much higher density of large cells than the stroma. Tenofovir partitions into these cells, where it is phosphorylated into its bioactive form, tenofovir diphosphate. However, the hydrophilic nature of tenofovir reduces its ability to readily cross the lipid-rich cell membranes, thus slowing down the overall diffusion rate of tenofovir in the epithelium. In contrast, the stroma has a much lower cell density and possesses hydrophilic pathways within its collagen matrix. Thence, the stromal environment overall is more hydrophilic than the epithelium and offers less resistance to the transport of hydrophilic molecules such as tenofovir. However, this could hinder transport of lipophilic microbicide drugs (e.g., dapivirine).

Although this study was conducted on normal, non-inflamed tissue, it should be noted that inflammation could alter mucosal composition, structure, and function, and thus the transport rates of tissue. The tissue may experience swelling and its epithelial structural integrity could be compromised [44]. Additional studies could explore the consequences of tissue inflammation on drug transport properties.

The methodology here for measuring and characterizing drug delivery into tissue could potentially be implemented on commercial confocal RS instruments without integrated OCT subsystems. Epithelial and stromal tissues have unique Raman spectral features (Fig 6A) that can be used to determine the thicknesses of the layers on those instruments. By scanning in depth through the sample and applying an appropriate correction factor (e.g., Fig 2A, see the Instrumentation section), the spectroscopic transition could be observed and a rough estimate of the epithelial thickness could be determined. With this approach, care would need to be exercised to apply an appropriate correction factor, because this factor is dependent upon the optical elements in the system and the optical properties of the tissue, which vary significantly over different tissue types [45, 46].

Results here enable determination of key information about the drug delivery performance of a microbicide gel product. The derived transport parameters can be used to compare drug and vehicle systems, contributing to improved product performance evaluation and pre-clinical development. These parameters are important inputs to deterministic computational microbicide PK models [10, 15, 16]. Such models relate product properties of the vehicle and drug, details of the dosage regimen, and properties of the host environment, to spatiotemporal drug concentration distributions in target compartments. This is valuable information in understanding the determinants of product prophylactic functionality, and in using that knowledge to improve product and dosage regimen design.

## Supporting information

S1 Table Tenofovir Raman measurements vs. tenofovir concentrations. (DOCX)

S2 Table Transport parameters derived from 6 independent experiments. (DOCX)

## References

[1] Neves Jd, Sarmento B. Drug delivery and development of anti-HIV microbicides: Pan Stanford; 2014. 10.1016/j.addr.2016.01.017 26829288

[2] Karim QA, Karim SSA, Frohlich JA, Grobler AC, Baxter C, Mansoor LE, et al Effectiveness and Safety of Tenofovir Gel, an Antiretroviral Microbicide, for the Prevention of HIV Infection in Women. Science. 2010;329(5996):1168–74. doi: 10.1126/science.1193748 WOS:000281485600026. 20643915 10.1126/science.1193748 PMC3001187

[3] McGowan I, Hoesley C, Cranston RD, Andrew P, Janocko L, Dai JY, et al A phase 1 randomized, double blind, placebo controlled rectal safety and acceptability study of tenofovir 1% gel (MTN-007). PloS one. 2013;8(4):e60147 doi: 10.1371/journal.pone.0060147 23573238 10.1371/journal.pone.0060147 PMC3616022

[4] Baeten JM, Palanee-Phillips T, Brown ER, Schwartz K, Soto-Torres LE, Govender V, et al Use of a Vaginal Ring Containing Dapivirine for HIV-1 Prevention in Women. New England Journal of Medicine. 2016. 10.1056/NEJMoa1506110 PMC4993693 26900902

[5] Lai BE, Geonnotti AR, DeSoto MG, Montefiori DC, Katz DF. Semi-solid gels function as physical barriers to human immunodeficiency virus transport in vitro. Antiviral Research. 2010;88(2):143–51. doi: 10.1016/j.antiviral.2010.08.006 WOS:000284680100002. 20709109 10.1016/j.antiviral.2010.08.006 PMC3072786

[6] Marrazzo J, Ramjee G, Nair G, Palanee T, Mkhize B, Nakabiito C, et al., editors. Pre-exposure prophylaxis for HIV in women: daily oral tenofovir, oral tenofovir/emtricitabine, or vaginal tenofovir gel in the VOICE study (MTN 003). 20th Conference on Retroviruses and Opportunistic infections; 2013: Atlanta GA.

[7] Rees H, S. Delany-Moretlwe, D. Baron, C. Lombard, G. Gray, L. Myer, R. Panchia, J. Schwartz, and G. Doncel., editor FACTS 001 phase III trial of pericoital tenofovir 1% gel for HIV prevention in women. Conference on Retroviruses and Opportunistic Infections (CROI); 2015; Seattle, WA.

[8] Marrazzo JM, Ramjee G, Richardson BA, Gomez K, Mgodi N, Nair G, et al Tenofovir-Based Preexposure Prophylaxis for HIV Infection among African Women. New England Journal of Medicine. 2015;372(6):509–18. doi: 10.1056/NEJMoa1402269 . 25651245 10.1056/NEJMoa1402269 PMC4341965

[9] USAID. FACTS 001 Trial: Questions and Answers2015. http://www.usaid.gov/sites/default/files/documents/1864/FACTS-001.pdf.

[10] Gao Y, Katz DF. Multicompartmental pharmacokinetic model of tenofovir delivery by a vaginal gel. PLoS One. 2013;8(9):e74404 doi: 10.1371/journal.pone.0074404 . 24040241 10.1371/journal.pone.0074404 PMC3770582

[11] Gao Y, Katz DF. Multicompartmental Pharmacokinetic Model of Tenofovir Delivery to the Rectal Mucosa by an Enema. PloS one. 2017;12(1):e0167696 doi: 10.1371/journal.pone.0167696 28114388 10.1371/journal.pone.0167696 PMC5256988

[12] Franz TJ. Percutaneous Absorption—Relevance of In vitro Data. Journal of Investigative Dermatology. 1975;64(3):190–5. doi: 10.1111/1523-1747.Ep12533356 WOS:A1975V973900011. 123263 10.1111/1523-1747.ep12533356

[13] Rohan LC, Moncla BJ, Kunjara Na Ayudhya RP, Cost M, Huang Y, Gai F, et al In vitro and ex vivo testing of tenofovir shows it is effective as an HIV-1 microbicide. PLoS One. 2010;5(2):e9310 doi: 10.1371/journal.pone.0009310 . 20174579 10.1371/journal.pone.0009310 PMC2824823

[14] Dezzutti CS, Rohan LC, Wang L, Uranker K, Shetler C, Cost M, et al Reformulated tenofovir gel for use as a dual compartment microbicide. Journal of Antimicrobial Chemotherapy. 2012;67(9):2139–42. doi: 10.1093/jac/dks173 22581908 10.1093/jac/dks173 PMC3417689

[15] Katz DF, Yuan A, Gao Y. Vaginal drug distribution modeling. Advanced drug delivery reviews. 2015;92:2–13. doi: 10.1016/j.addr.2015.04.017 25933938 10.1016/j.addr.2015.04.017 PMC4600641

[16] Gao Y, Yuan A, Chuchuen O, Ham A, Yang KH, Katz DF. Vaginal deployment and tenofovir delivery by microbicide gels. Drug Deliv Transl Res. 2015;5(3):279–94. doi: 10.1007/s13346-015-0227-1 . 25874971 10.1007/s13346-015-0227-1 PMC4420798

[17] Nel AM, Coplan P, Smythe SC, McCord K, Mitchnick M, Kaptur PE, et al Pharmacokinetic Assessment of Dapivirine Vaginal Microbicide Gel in Healthy, HIV-Negative Women. Aids Research and Human Retroviruses. 2010;26(11):1181–90. doi: 10.1089/aid.2009.0227 WOS:000284311900004. 20854207 10.1089/aid.2009.0227

[18] Hendrix CW, Chen BA, Guddera V, Hoesley C, Justman J, Nakabiito C, et al MTN-001: randomized pharmacokinetic cross-over study comparing tenofovir vaginal gel and oral tablets in vaginal tissue and other compartments. PLoS One. 2013;8(1):e55013 doi: 10.1371/journal.pone.0055013 . 23383037 10.1371/journal.pone.0055013 PMC3559346

[19] Roy K, Bottrill I, Ingrams DR, Pankratov MM, Rebeiz EE, Woo P, et al., editors. Diagnostic fluorescence spectroscopy of oral mucosa. Photonics West'95; 1995: International Society for Optics and Photonics.

[20] Wood BR, Quinn MA, Burden FR, McNaughton D. An investigation into FTIR spectroscopy as a biodiagnostic tool for cervical cancer. Biospectroscopy. 1996;2(3):143–53.

[21] Xiao C, Moore DJ, Rerek ME, Flach CR, Mendelsohn R. Feasibility of tracking phospholipid permeation into skin using infrared and Raman microscopic imaging. Journal of investigative dermatology. 2005;124(3):622–32. doi: 10.1111/j.0022-202X.2004.23608.x 15737204 10.1111/j.0022-202X.2004.23608.x

[22] Tfayli A, Piot O, Pitre F, Manfait M. Follow-up of drug permeation through excised human skin with confocal Raman microspectroscopy. European Biophysics Journal. 2007;36(8):1049–58. doi: 10.1007/s00249-007-0191-x 17565493 10.1007/s00249-007-0191-x

[23] Chuchuen O, Henderson MH, Sykes C, Kim MS, Kashuba AD, Katz DF. Quantitative analysis of microbicide concentrations in fluids, gels and tissues using confocal Raman spectroscopy. PLoS One. 2013;8(12):e85124 doi: 10.1371/journal.pone.0085124 . 24386455 10.1371/journal.pone.0085124 PMC3875564

[24] Maher JR, Chuchuen O, Henderson MH, Kim S, Rinehart MT, Kashuba AD, et al Co-localized confocal Raman spectroscopy and optical coherence tomography (CRS-OCT) for depth-resolved analyte detection in tissue. Biomed Opt Express. 2015;6(6):2022–35. doi: 10.1364/BOE.6.002022 . 26114026 10.1364/BOE.6.002022 PMC4473741

[25] Izatt JA, Kulkarni MD, Kobayashi K, Sivak MV, Barton JK, Welch AJ. Optical coherence tomography for biodiagnostics. Optics and Photonics News. 1997;8(5):41.

[26] Schmitt JM. Optical coherence tomography (OCT): a review. Selected Topics in Quantum Electronics, IEEE Journal of. 1999;5(4):1205–15. 10.1109/2944.796347 PMC4358303 25774083

[27] Fujimoto JG, Brezinski ME, Tearney GJ, Boppart SA, Bouma B, Hee MR, et al Optical biopsy and imaging using optical coherence tomography. Nature medicine. 1995;1(9):970–2. 7585229 10.1038/nm0995-970

[28] Belsey NA, Garrett NL, Contreras-Rojas LR, Pickup-Gerlaugh AJ, Price GJ, Moger J, et al Evaluation of drug delivery to intact and porated skin by coherent Raman scattering and fluorescence microscopies. Journal of Controlled Release. 2014;174:37–42. doi: 10.1016/j.jconrel.2013.11.002 24231405 10.1016/j.jconrel.2013.11.002

[29] Camp CH Jr, Cicerone MT. Chemically sensitive bioimaging with coherent Raman scattering. Nature Photonics. 2015;9(5):295–305.

[30] Squier CA, Mantz MJ, Schlievert PM, Davis CC. Porcine vagina ex vivo as a model for studying permeability and pathogenesis in mucosa. Journal of pharmaceutical sciences. 2008;97(1):9–21. doi: 10.1002/jps.21077 17721937 10.1002/jps.21077

[31] Davis CC, Baccam M, Mantz MJ, Osborn TW, Hill DR, Squier CA. Use of porcine vaginal tissue ex-vivo to model environmental effects on vaginal mucosa to toxic shock syndrome toxin-1. Toxicol Appl Pharmacol. 2014;274(2):240–8. doi: 10.1016/j.taap.2013.11.021 . 24333258 10.1016/j.taap.2013.11.021

[32] Van der Bijl P. Effect of freezing on the permeability of human buccal and vaginal mucosa. 1998.

[33] Mahalingam A, Simmons AP, Ugaonkar SR, Watson KM, Dezzutti CS, Rohan LC, et al Vaginal Microbicide Gel for Delivery of IQP-0528, a Pyrimidinedione Analog with a Dual Mechanism of Action against HIV-1. Antimicrobial Agents and Chemotherapy. 2011;55(4):1650–60. doi: 10.1128/AAC.01368-10 WOS:000288594600041. 21245437 10.1128/AAC.01368-10 PMC3067148

[34] Zidan AS, Habib MJ. Maximized mucoadhesion and skin permeation of anti-AIDS-loaded niosomal gels. J Pharm Sci. 2014;103(3):952–64. doi: 10.1002/jps.23867 . 24464823 10.1002/jps.23867

[35] Everall NJ. Confocal Raman microscopy: common errors and artefacts. Analyst. 2010;135(10):2512–22. doi: 10.1039/c0an00371a WOS:000282003800004. 20725670 10.1039/c0an00371a

[36] Berger AJ, Feld MS. Analytical method of estimating chemometric prediction error. Applied spectroscopy. 1997;51(5):725–32.

[37] Shrivastava A, Gupta V. Methods for the determination of limit of detection and limit of quantitation of the analytical methods. Chronicles of Young Scientists. 2011;2(1):21-.

[38] Haaland DM, Thomas EV. Partial least-squares methods for spectral analyses. 1. Relation to other quantitative calibration methods and the extraction of qualitative information. Analytical Chemistry. 1988;60(11):1193–202.

[39] Chuchuen O, Maher JR, Simons MG, Peters JJ, Wax AP, Katz DF. Label-Free Measurements of Tenofovir Diffusion Coefficients in a Microbicide Gel Using Raman Spectroscopy. Journal of Pharmaceutical Sciences. 2017;106(2):639–44. doi: 10.1016/j.xphs.2016.09.030 27837968 10.1016/j.xphs.2016.09.030 PMC5503201

[40] ur Rehman I, Movasaghi Z, Rehman S. Vibrational spectroscopy for tissue analysis: CRC Press; 2012.

[41] Severcan F, Haris PI. Vibrational spectroscopy in diagnosis and screening: IOS Press; 2012.

[42] Nguyen T, Gobinet C, Feru J, Pasco SB, Manfait M, Piot O. Characterization of type I and IV collagens by Raman microspectroscopy: Identification of spectral markers of the dermo-epidermal junction. Journal of Spectroscopy. 2012;27(5–6):421–7.

[43] Truskey GA, Yuan F, Katz DF. Transport phenomena in biological systems: Pearson/Prentice Hall Upper Saddle River NJ; 2004.

[44] Vargas G, Shilagard T, Johnston R, Bell B, Stegall RL, Vincent K, et al Use of high-resolution confocal imaging of the vaginal epithelial microstructure to detect microbicide toxicity. The Journal of infectious diseases. 2009;199(10):1546–52. doi: 10.1086/598221 19355817 10.1086/598221 PMC4503316

[45] Jacques SL. Optical properties of biological tissues: a review. Phys Med Biol. 2013;58(11):R37–61. doi: 10.1088/0031-9155/58/11/R37 . 23666068 10.1088/0031-9155/58/11/R37

[46] Cheong W-F, Prahl SA, Welch AJ. A review of the optical properties of biological tissues. IEEE journal of quantum electronics. 1990;26(12):2166–85.

