# Supplementary material for: Label-free analysis of tenofovir delivery to vaginal tissue using co-registered confocal Raman spectroscopy and optical coherence tomography
Source: PLoS One. 2017 Sep 29;12(9):e0185633. doi: 10.1371/journal.pone.0185633 (PMC5621692; doi:10.1371/journal.pone.0185633)
Supplement: S1 Table — (DOCX) [file pone.0185633.s001.docx]

S1 Table: Tenofovir Raman measurements vs. tenofovir concentrations

| **Concentration**  **(% w/w)** | **Raman measurements (a.u.)** | | | | | |
| --- | --- | --- | --- | --- | --- | --- |
|  | **1** | **2** | **3** | **4** | **5** | **6** |
| 0.00 | 0.00 | 0.00 | 0.00 | 0.00 | - | - |
| 0.27 | 2.38 | 2.40 | 2.17 | 1.67 | 2.09 | - |
| 0.37 | 3.58 | 3.00 | 2.68 | 3.00 | 3.24 | - |
| 0.45 | 3.49 | 3.98 | 4.15 | 3.93 | 3.48 | 4.11 |
| 0.56 | 5.20 | 5.06 | 4.79 | 4.73 | 4.95 | - |
| 0.71 | 6.26 | 6.34 | 6.72 | 5.27 | 6.24 | - |
| 0.87 | 7.29 | 6.62 | 6.81 | 7.56 | 6.81 | - |
| 0.96 | 7.83 | 7.57 | 7.76 | 7.24 | 7.32 |  |
| 0.98 | 7.86 | 8.16 | 8.72 | 9.06 | 9.78 | - |
| 1.14 | 8.87 | 10.37 | 9.44 | 8.72 | 8.70 | - |
| 1.27 | 11.06 | 11.39 | 11.47 | 10.64 | 11.14 | - |
| 1.44 | 13.26 | 10.30 | 12.59 | 12.28 | 11.46 | - |
