# Supplementary material for: Label-free analysis of tenofovir delivery to vaginal tissue using co-registered confocal Raman spectroscopy and optical coherence tomography
Source: PLoS One. 2017 Sep 29;12(9):e0185633. doi: 10.1371/journal.pone.0185633 (PMC5621692; doi:10.1371/journal.pone.0185633)
Supplement: S2 Table — (DOCX) [file pone.0185633.s002.docx]

S2 Table: Transport parameters derived from 6 independent experiments

| Exp. # | D_EP_ (cm^2^/s) | D_ST_ (cm^2^/s) | Φ_EP/G_ | Φ_ST/EP_ |
| --- | --- | --- | --- | --- |
| 1 | 6.03 x 10^-8^ | 1.22 x 10^-7^ | 0.38 | 1.33 |
| 2 | 5.04 x 10^-8^ | 4.14 x 10^-7^ | 0.57 | 1.01 |
| 3 | 8.56 x 10^-8^ | 4.64 x 10^-7^ | 0.81 | 1.04 |
| 4 | 2.71 x 10^-8^ | 5.92 x 10^-7^ | 0.43 | 1.41 |
| 5 | 6.18 x 10^-8^ | 4.60 x 10^-7^ | 0.45 | 1.09 |
| 6 | 8.08 x 10^-8^ | 6.57 x 10^-7^ | 0.56 | 1.14 |
